# Genetic and epigenetic characteristics in ovarian tissues from polycystic ovary syndrome patients with irregular menstruation resemble those of ovarian cancer

**DOI:** 10.1186/s12902-019-0356-5

**Published:** 2019-03-12

**Authors:** Jiao Jiao, Matthew Sagnelli, Bei Shi, Yuanyuan Fang, Ziqi Shen, Tianyu Tang, Bingying Dong, Da Li, Xiuxia Wang

**Affiliations:** 1 Center of Reproductive Medicine, Shengjing Hospital of China Medical University, Shenyang, 110004 China; 2 University of Connecticut School of Medicine, Farmington, CT 06030 USA; 3 Department of Physiology, College of Life Science, China Medical University, Shenyang, 110122 China; 4 Functional Laboratory Center, College of Basic Medical Science, China Medical University, Shenyang, 110122 China

**Keywords:** PCOS, Irregular menstruation, Ovarian Cancer, *BRCA1*, *MLH1*

## Abstract

**Background:**

Irregular menstruation is clinically associated with an increased risk for ovarian cancer and disease-related mortality. This relationship remains poorly understood, and a mechanism explaining it has yet to be described.

**Methods:**

Ovarian tissues from women with polycystic ovary syndrome (PCOS) and regular menstruation (*n* = 10) or irregular menstruation (n = 10) were subjected to DNA methylation sequencing, real-time PCR array, whole-exome sequencing, and bioinformatics analysis.

**Results:**

We demonstrated that ovarian tissue from PCOS patients with irregular menstruation displayed global DNA hypomethylation, as well as hypomethylation at several functionally and oncologically significant regions. Furthermore, we showed that several cancer-related genes were aberrantly expressed in ovarian tissue from patients with irregular menstruation, and that their mRNA and microRNA profiles shared appreciable levels of coincidence with those from ovarian cancer tissue. We identified multiple point mutations in both the BRCA1 and MLH1 genes in patients with irregular menstruation, and predicted the potential pathogenicity of these mutations using bioinformatics analyses.

**Conclusions:**

Due to the nature of ovarian cancer, it is important to broaden our understanding of the pathogenesis and risk factors of the disease. Herein, we provide the first description of a genetic and epigenetic basis for the clinical relationship between irregular menstruation and an increased risk for ovarian cancer.

**Electronic supplementary material:**

The online version of this article (10.1186/s12902-019-0356-5) contains supplementary material, which is available to authorized users.

## Background

Ovarian cancer is associated with high morbidity and low 5-year survival rates [1]. The trend for poor outcomes in ovarian cancer is due in part to the fact that 60% of patients first present with the disease once it has reached an advanced distant stage, at which the 5-year survival rate is 29% [1], highlighting the importance of understanding risk factors to facilitate early detection and intervention. The carcinogenesis of ovarian cancer is hormonally driven. Notably, an increased number of ovulatory cycles confers an elevated risk for ovarian cancer [2]; however, a relationship between irregularly long menstrual periods (low number of cycles) and an increased risk of ovarian cancer incidence and mortality has also been described [3].

Polycystic ovary syndrome (PCOS) is a collection of symptoms arising from an excess of androgens. Menstrual cycle-specific symptoms of PCOS include amenorrhea, oligomenorrhea, or menorrhagia, with the most common feature being irregularly long menstrual periods. The link between PCOS, menstrual period length, and ovarian cancer remains unclear; several studies have provided evidence both supporting and refuting an association [3, 4], but few large population studies have investigated this relationship.

The association between ovarian cancer and PCOS remains controversial with various studies yielding mixed results and lacking statistical power. A 2016 review found studies on the relationship between PCOS and ovarian cancer with risk ratios ranging from 1.0–2.4, none of which were statistically significant [5]. A 1996 study reported a positive relationship between PCOS and epithelial ovarian cancer (odds ratio [OR] 2.5; 95% confidence interval [CI] 1.1–5.9) [6]. Interestingly, that study also found that never having used oral contraceptives (OR 10.5; 95% CI 2.5–44.2) and a low body mass index at age 18 years (OR 15.6; 95% CI 3.4–71.0) strengthened the association between PCOS and ovarian cancer [6]. A 2014 meta-analysis found no association between PCOS and ovarian cancer (OR 1.41; 95% CI 0.93–2.1), but detected a positive association between PCOS and ovarian cancer in women < 54 years (OR 2.52; 95% CI 1.08–5.89) [7]. Current literature gives no clear consensus on the association between these two conditions, and suggests that the relationship is multifactorial and stratified among different subtypes of ovarian cancer. These findings highlight the importance of continued investigation into the relationship between ovarian cancer and PCOS.

The genetic and molecular characteristics of ovarian cancer and PCOS have been well described. Ovarian cancer exhibits many hallmark cancer characteristics, such as DNA hypomethylation and aberrant expression of a number of critical oncogenes and tumor suppressor genes. Additionally, dozens of microRNAs (miRNAs) have been associated with tumorigenesis and progression of ovarian cancer [8, 9]. The genetic basis of PCOS is beginning to become clear, and numerous genes have been associated with the condition. Many of the genes implicated in PCOS are involved in hormonal pathways and functioning, such as gonadotropin action [10]. The genetics resulting in the irregular menstruation phenotype are less clear than the aforementioned, as menstrual cycle characteristics are influenced by both environmental and genetic factors [11]; however, polymorphisms in specific genes have been identified in patients with irregular menstruation [12]. Much remains to be understood about the genetics of irregular menstruation, particularly in the context of its relationship to ovarian cancer.

While it has been demonstrated that clinically irregular menstruation is associated with an increased risk for ovarian cancer, there has yet to be a molecular mechanism describing this relationship. Furthermore, PCOS and irregular menstruation are closely intertwined; thus, the association between these two clinical conditions and ovarian cancer needs to be distinguished at a molecular level. We sought to address these questions by analyzing ovarian tissue samples from two groups of patients with PCOS: patients with regular menstruation (short cycles) and patients with irregular menstruation (long cycles). Herein, we investigated the differences in DNA methylation, mRNA expression, and miRNA expression profiles between the two groups, and we utilized whole-exome sequencing to search for mutations in critical oncogenic genes in both groups.

## Methods

### Ethical statement

This study was conducted in accordance with the ethical standards outlined in the Helsinki Declaration of 1975.

### Patients and tissue collection

This study was approved by the Institutional Review Board of Shengjing Hospital, China Medical University (reference number 2015PS108K). All participants provided written informed consent. A total of 20 patients with PCOS < 35 years (10 regular menstruation and 10 irregular menstruation) were recruited to participate; recruitment took place from February 2014 to December 2015. Hormonal assays were performed during the first 5 days of spontaneous menstrual cycles or progestin-withdrawal bleeding. PCOS ovarian tissue was obtained during the follicular phase via a laparoscopic wedge resection 7–11 days after the hormonal evaluation. PCOS was diagnosed according to the 2003 Rotterdam criteria. The exclusion criteria were: tobacco smoking, hormonal medication; medications (insulin-sensitizing drugs, oral contraceptives, antiandrogens, corticosteroids, and gonadotropin releasing hormone agonists or antagonists) taken within the preceding 3 months; and a history of any known neoplasm, infectious, or inflammatory disease. None of the participants had a family history of PCOS or ovarian cancer. Cycles within the range of 27–35 days were defined as regular menstruation, and cycles > 2 months were defined as irregular menstruation. All 10 patients in the irregular menstruation group had cycles of 2–12 months. The subjects’ characteristics are provided in Table 1.

**Table 1:** Description of the study participants

	Regular menstruation (27–35 days)	Irregular menstruation (2–12 months)	*p-*value
Age (year)	24.5 ± 3.2	25.8 ± 4.1	NS
BMI (kg/m^2^)	29.7 ± 2.9	30.3 ± 3.2	NS
Total testosterone (nmol/liter)	1.75 ± 0.28	1.77 ± 0.33	NS
FSH (IU/liter)	4.7 ± 1.4	5.3 ± 1.2	NS
LH (IU/liter)	11.6 ± 3.4	12.6 ± 4.8	NS
FPG (mmol/liter)	5.6 ± 0.6	5.6 ± 0.8	NS
FI (mIU/liter)	37.2 ± 26.2	39.4 ± 18.7	NS

Abbreviations: *BMI*, body mass index; *FSH*, follicle-stimulating hormone; *LH*, luteinizing hormone; *FPG*, fasting plasma glucose; *FI*, fasting serum insulin; *PCOS*, polycystic ovarian syndrome. Mean ± SD. NS, not significant. Significant difference between regular and irregular menstruation after independent-sample *t*-test

### DNA methylation sequencing

Genomic DNA of the ovarian tissues were extracted using the DNeasy Blood and Tissue kit (Qiagen, Valencia, CA, USA) following the manufacturer’s instructions and sent to the Novogene Corp. (Beijing, China) for bisulfite treatment and library preparation. After cluster generation, the library preparations were sequenced on an Illumina Hiseq 2000/2500 platform (Illumina Inc. San Diego, CA, USA), and 125/150 bp paired-end reads were generated. Image analysis and base calling were performed with the Illumina CASAVA pipeline, and 125/150 bp paired-end reads were generated. After removing the low-quality reads, Bismark software (version 0.16.3) was used to perform alignments of the bisulfite-treated reads to a reference genome with the default parameters. The reference genome was first transformed to bisulfite-converted version (C-to-T and G-to-A converted) and then indexed using bowtie2. Sequence reads were also transformed into full bisulfite-converted versions (C-to-T and G-to-A converted) before they were aligned to similarly converted versions of the genome in a directional manner. Read pairs that shared the same coordinates in the genome were regarded duplicates and were removed before methylation state calling, thus avoiding potential methylation level calculation bias. The bisulfite non-conversion rate was calculated as the percentage of cytosines sequenced at cytosine reference positions in the lambda genome. We used Picard tools (https://github.com/broadinstitute/picard, version 2.2.1) to further process the aligned data files. In particular, we used CollectHsMetrics to calculate all hybrid capture-related metrics, including the on target rate and fold enrichment. Mean and median coverage metrics of the target regions were calculated with Picard tools. Single nucleotide polymorphism (SNP) calling was performed with Bis-SNP. Bisulfite treatment converts unmethylated C residues to T, whereas methylated C residues are not converted. Therefore, the distinction between the alternate allele and bisulfite conversion is not possible for C/T and G/A SNPs (depending on the strand). However, because Agilent SureSelect captures negative stranded DNA fragments, only G/A SNPs needed to be filtered out. The SNPs were annotated by snpEff software (version 4.2). Methylation values were obtained using the Bis-SNP program with default parameters. Only methylation sites that were on the negative strand were retained. CpGs were included in subsequent distribution analysis if the number of sequence reads was five or greater. Sites that were on-target were determined using the bedtools intersect command.

We also estimated the DNA methylation level of different genomic regions, including the promoter, 5′untranslated region (UTR), exons, introns, 3′UTR, intergenic regions, CGI regions, CGI shore, and repetitive regions (especially the LINE-1 region). Promoters were further classified as high-density CpG promoters and low-density CpG promoters based on corresponding CpG density. The DNA methylation level of these genomic regions was calculated based on the average methylation level of all covered CpG sites within these regions. Sequencing data were deposited with the NCBI Sequence Read Archive (SRA) under accession number PRJNA485029.

### Genetic studies

The 23 genes known to be mutated in ovarian cancer, can be found at the NIH-Genetics Home Reference (https://ghr.nlm.nih.gov/condition/ovarian-cancer#genes) and were analyzed by whole-exome sequencing. Whole-exome capture (Agilent SureSelect Human All Exon Kit; Agilent Technologies, Palo Alto, CA, USA) was performed using the standard protocols. The clustering of the index-coded samples was performed on a cBot Cluster Generation System using Hiseq PE Cluster Kit (Illumina). After cluster generation, the DNA libraries were sequenced on the Illumina Hiseq platform, and 150 bp paired-end reads were generated. Valid sequencing data were mapped to the reference human genome (UCSC hg19) using the Burrows-Wheeler Aligner to obtain the original mapping results stored in BAM format. Samtools mpileup and bcftools were used for variant calling and to identify SNPs and indels. dbSNP, 1000 Genome, and other related existing databases were applied to characterize the detected variants. Given the significance of exonic variants, gene transcript annotation databases, such as Consensus CDS, RefSeq, Ensembl, and UCSC, were also included to determine alternating amino acids. The somatic SNV was detected by muTect, and the somatic indels were detected with Strelka. Control-FREEC was used to detect somatic CNV. Sanger sequencing was performed to confirm the variants found by whole-exome sequencing. The functional prediction was performed by the PROVEAN, SIFT, LRT, MutationTaster2, and PolyPhen-2 programs.

### Real-time PCR array

Total RNA was extracted using Trizol reagent (Invitrogen, Carlsbad, CA, USA) according to the manufacturer’s protocol. DNA contamination was removed by adding DNase I (Invitrogen). The MystiCq microRNA qPCR Assay (Sigma, St. Louis, MO, USA) was used according to the manufacturers’ instructions to quantify the 84 human ovarian cancer-related miRNAs. The indicated miRNA levels were normalized against U6. These 84 miRNAs are potential biomarkers of ovarian cancer progression, and have been followed up with functional studies, which have identified potential specific oncogenic mechanisms (Ovarian Cancer miScript miRNA PCR Array, QIAGEN, Valencia, CA, USA). To quantify the 66 cancer-related genes, total RNA was reverse-transcribed from 2 μg RNA using the PrimeScript™ RT Reagent Kit with the gDNA Eraser (TaKaRa, Dalian, China) and amplified with the GoTaq® qPCR Master Mix (Promega, Madison, WI, USA) on the ABI ViiA 7 Real-time PCR system (Applied Biosystems, Foster City, CA, USA). Specificity was verified by a melting curve analysis and agarose gel electrophoresis. The threshold cycle (Ct) values of each sample were used for the post-PCR data analysis. Gene expression levels were normalized against GAPDH. The specific primer sequences are listed in Additional file 1. These 66 genes were representative of the six biological pathways involved in transformation and tumorigenesis. 1) Angiogenesis: ANGPT1, ANGPT2, CCL2, FGF2, KDR, PGF, SERPINF1, TEK, and VEGFC; 2) Apoptosis: BCL2L11, BIRC3, CASP2, CASP7, CASP9, CFLAR, FASLG, NOL3, and XIAP; 3) Cell Cycle: AURKA, CCND3, CDC20, E2F4, MCM2, SKP2, STMN1, and WEE1; 4) Cell Senescence: BMI1, IGFBP3, IGFBP5, MAPK14, SOD1, and TBX2; 5) DNA Damage & Repair: DDB2, DDIT3, ERCC3, ERCC5, GADD45G, LIG4, and PPP1R15A; 6) Epithelial-to-Mesenchymal Transition: FOXC2, GSC, KRT14, OCLN, SNAI1, SNAI2, SNAI3, and SOX10; 7) Hypoxia: ADM, ARNT, CA9, HMOX1, and LDHA; 8) Metabolism: ACLY, ATP5A1, CPT2, G6PD, GPD2, LPL, PFKL, and UQCRFS1; and 9) Telomeres & Telomerase: DKC1, PINX1, TERF1, TERF2IP, TINF2, and TNKS2.

### Mapping the gene expression trends in ovarian cancer using the TCGA and GEO databases

A total of 379 ovarian serous cystadenocarcinoma and 46 normal ovary tissue samples were used for the integrated analysis. The 379 ovarian serous cystadenocarcinoma RNAseq data were downloaded from The Cancer Genome Atlas (TCGA) database using the Data Transfer Tool (provided by GDC Apps) (https://portal.gdc.cancer.gov/). Those sequence data were derived from the Affymetrix Human Genome U133 Plus 2.0 Array platform. Our research meets publication guidelines provided by TCGA (https://www.cancer.gov/about-nci/organization/ccg/research/structural-genomics/tcga/using-tcga/citing-tcga). The 46 normal ovarian tissues were downloaded from the Gene Expression Omnibus (GEO) (https://www.ncbi.nlm.nih.gov/geo/) database. The detailed information of the 46 normal ovarian tissue samples is listed in Additional file 2. Those sequencing data were also derived from same platform with 379 ovarian cancer samples. We merged those data using R codes according to the gene symbols. Differential expression of RNAs was identified using the “edgeR” bioconductor package in R, with thresholds of |log2FC| *>* 1.0 and an adjusted *p*-value *<* 0.05. Finally, we plotted heatmaps of the selected genes using the “pheatmap” package in R.

### Statistical analysis

Data are presented as mean ± standard deviation. Statistical differences in the data were evaluated by Student’s *t*-test or one-way analysis of variance as appropriate, and were considered significant at *p* < 0.05.

## Results

### Global DNA hypomethylation in the irregular menstruation group

**Fig. 1:**
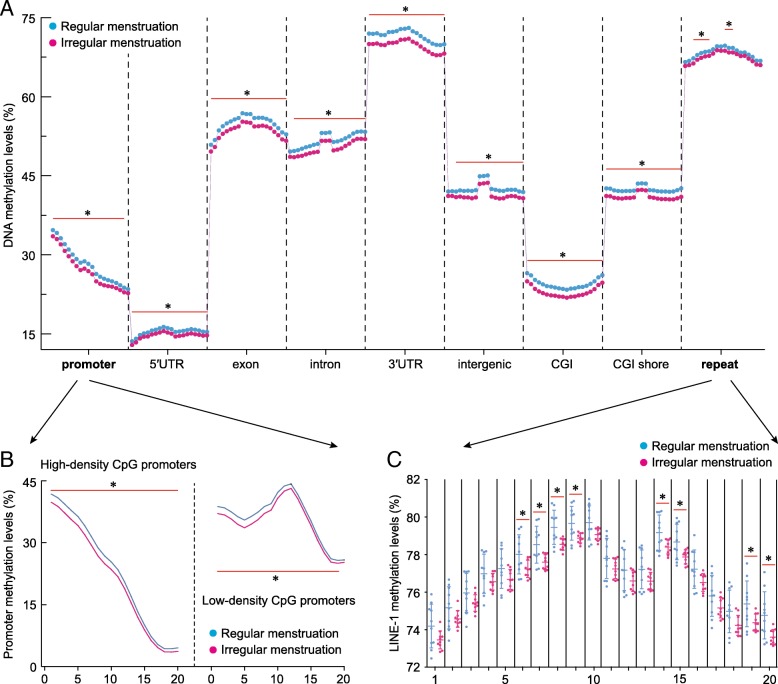
DNA methylation dynamics on different annotated genomic elements. **a** Differences in methylation levels of the promoter, 5′UTR, exon, intron, 3′UTR, intergenic, CGI, CGI shore, and repeat regions in human ovarian tissues from women with regular and irregular menstrual cycles. DNA methylation level was calculated as the average DNA methylation level based on all CpG sites in each region, and each region was further divided into 20 bins (20 dots) to evaluate the methylation levels. **b** Promoters were further classified as high-density CpG promoters and low-density CpG promoters. **c** Differences in methylation levels of the long interspersed nuclear element (LINE-1) were further analyzed. Each group, *n* = 10. *p < 0.05 vs. control group

Ovarian tissues from the irregular menstruation group demonstrated whole-genome hypomethylation compared to those in the regular menstruation group (Fig. 1a). To more closely examine the distribution of this trend across the genome and its functional significance, we examined DNA methylation at several biologically important sites. Promoter methylation was significantly lower in the irregular menstruation group compared to the control; both high and low-CpG density promoters, two groups that differ in their rate of mutation [13], showed the same trend (Fig. 1b). We further examined intronic and extronic regions, the 5′ and 3′UTRs, intergenic region, as well as CpG islands (CGI) and CGI shores; all showed hypomethylation in the irregular menstruation group compared to the control (Fig. 1a). Furthermore, we examined methylation of long interspersed nuclear element-1 (LINE-1). LINE-1 belongs to a family of transposons that have been implicated in the oncogenesis of a number of malignancies and have been shown to cause genomic instability [14, 15]. Our data showed significant hypomethylation at several sites along LINE-1 in the irregular menstruation group (Fig. 1c). Taken together, these results indicate global DNA hypomethylation, particularly at functionally important sites, in the irregular menstruation group.

### miRNA profiles in the irregular menstruation group mirror those of ovarian cancer

**Fig. 2:**
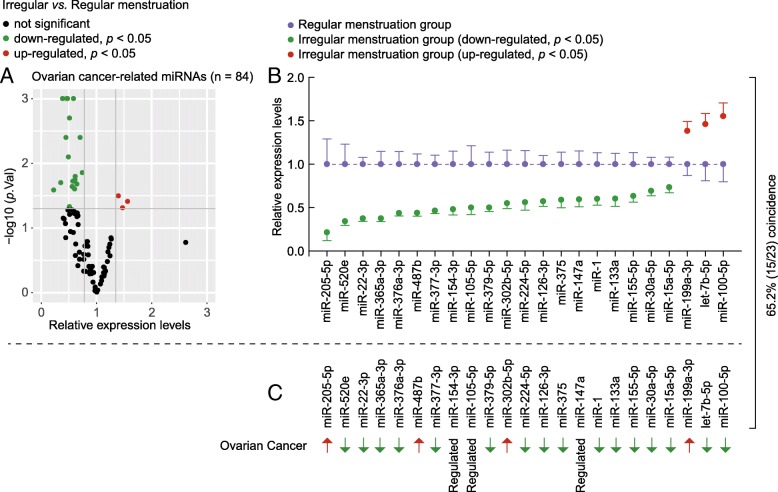
miRNA expression profiles in ovarian tissue from women with regular or irregular menstruation compared to tissues from ovarian cancer. Intracellular levels of ovarian cancer-related miRNAs in ovarian tissue from women with regular and irregular menstrual cycles. **a** Volcano plots depicting the relative expression levels (x-axis) and statistical significance (−log10 *p* value, y-axis) of ovarian cancer-related miRNAs in ovarian tissue from women with regular and irregular menstrual cycles. **b** Differences in the expression patterns of 23 ovarian cancer-related miRNAs in ovarian tissue from women with regular and irregular menstrual cycles. Each group, n = 10. **c** The differentially expressed 23 ovarian cancer-related miRNAs between ovarian cancer and normal ovarian tissues

We examined the expression levels of 84 ovarian cancer-related miRNAs in the ovarian tissues of the regular and irregular menstruation groups. We identified 23 miRNAs to be either significantly up- or down-regulated in the irregular menstruation group compared to the control group (Fig. 2a). Next, we compared the expression patterns of these 23 miRNAs to their respective patterns found in ovarian cancer and observed that the expression profiles of those 23 miRNAs shared a 65.2% coincidence between the irregular menstruation group and the ovarian cancer data (Fig. 2b and c). Many of these miRNAs have functionally significant roles in many oncogenic pathways, such as cell proliferation (miR-520e and miR-22-3p), cell migration (miR-520e) and the epithelial to mesenchymal transition (miR-205) [16–18]. These results highlight a marked similarity in the miRNA expression profiles of ovarian cancer and the irregular menstruation group.

### Cancer-related gene expression in the irregular menstruation group is similar to ovarian cancer

**Fig. 3:**
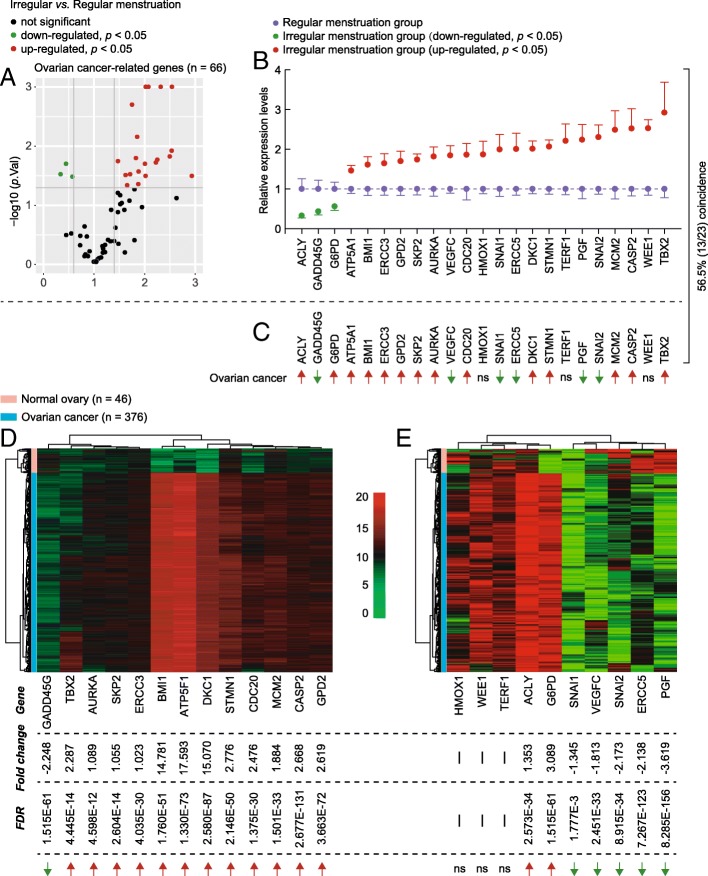
Expression levels of cancer-related genes in ovarian tissue from women with regular and irregular menstrual cycles. **a** Volcano plots depicting the relative expression levels (x-axis) and statistical significance (−log10 *p* value, y-axis) of cancer-related genes in ovarian tissue from women with regular and irregular menstrual cycles. **b** Differences in expression patterns of 23 cancer-related genes in ovarian tissue from women with regular and irregular menstrual cycles. Each group, n = 10. **c** Trends in the 23 differently expressed cancer-related genes in ovarian cancer. **d** and **e**. Heat-map showing the expression levels of the differentially expressed genes in ovarian cancer that were coincident (**d**) or mismatched (**e**) with the expression trend of the irregular menstruation group. FDR, false discovery rate. Ns, not significant

We surveyed the expression levels of 66 cancer-related genes in the ovarian tissue of the regular and irregular menstruation groups. We identified 23 genes to be differentially expressed in the irregular menstruation group compared to the control (Fig. 3a). To further examine the significance of these results as associated with ovarian cancer, we analyzed the coincidence of the expression levels of the 23 genes between the irregular menstruation group and ovarian cancer mRNA sequencing data, revealing a 56.5% match (Fig. 3b and c). To further confirm this result, we illustrated the expression trends of these 23 cancer-related genes in ovarian cancer coupled with their coincidence to the irregular menstruation group (Fig. 3d and e). Taken together, these results demonstrate an array of oncogenic and tumor suppressor genes in the irregular menstruation group that are expressed in a pattern similar to that found in ovarian cancers.

### Identification of *BRCA1* and *MLH1* mutations in the irregular menstruation group

**Fig. 4:**
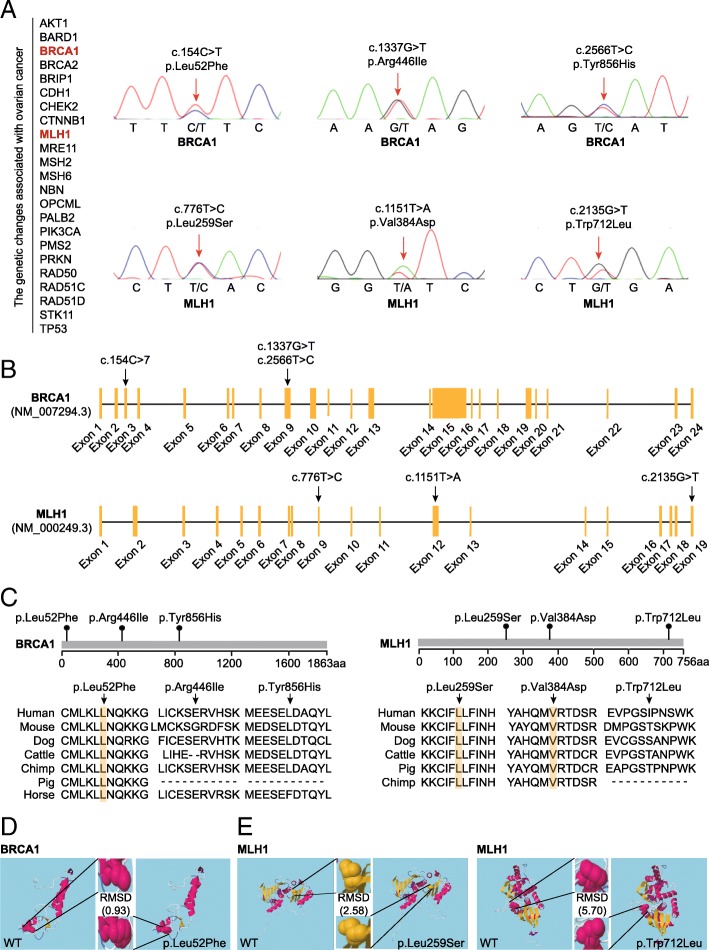
Identification of the *BRCA1* and *MLH1* mutations. **a** The genetic changes in 23 genes associated with ovarian cancer were analyzed by whole-exome sequencing. Sanger sequencing was performed to confirm the variants detected by whole-exome sequencing. **b** Locations and conservation of mutations in the *BRCA1* and *MLH1* genes. The positions of all mutations are indicated in the *BRCA1* and *MLH1* genomic structures. **c** Conservation of the mutated amino acids is indicated by the alignment of seven mammalian species. **d** Secondary structural modeling of the wild-type *BRCA1* and the p.Leu52Phe variant. **e** Secondary structural modeling of the wild-type *MLH1*, and the p.Leu259Ser and p.Trp712Leu variants. Modeling was performed using RaptorX (http://raptorx.uchicago.edu/). RMSD, root mean square deviation, which represents the average distance between atoms of the wild-type and mutant proteins

To gain more insight into the genetic relationship between ovarian cancer and menstruation, we analyzed the 23 genes known to be mutated in ovarian cancer that were identified as being differentially expressed in the irregular mensuration group compared to the control using whole-exome and Sanger sequencing. This method lead to the identification of three point mutations in the *BRCA1 and MLH1* genes in the irregular menstruation group (Fig. 4a). Each of these mutations was mapped in their respective genes; the three point mutations in the *BRCA1* gene were cytosine to thymine (C > T) in Exon 3, and G > T and T > C in Exon 9; the three point mutations in the *MLH1* gene were T > C in Exon 9, T > A in Exon 12, and G > T in Exon 19 (Fig. 4b). To determine the conservation of these mutations, amino acid sequence alignment was compared among seven mammalian species; the Leu52Phe (154C > T) mutation in the *BRCA1* gene, along with the Leu259Ser (776 T > C) and Val384Asp (1151 T > A) mutations in the *MLH1* gene were conserved across all species examined (Fig. 4c). We then considered the functional effects of these mutations on the final protein products of the *BRCA1* and *MLH1* genes. Using secondary structure modeling, we revealed that the Leu52Phe (154C > T) mutation in the *BRCA1* gene led to an alternation in the three-dimensional confirmation of the BRCA1 protein, (Fig. 4d). Analysis of the *MLH1* gene mutation variants demonstrated that the Leu259Ser (776 T > C) and Trp712Leu (2135G > T) mutations resulted in a significant change in the three-dimensional confirmation from the wild type MLH1 protein (Fig. 4e). Next, we employed multiple bioinformatics analyses to predict the potential pathogenicity of these mutations (Table 2). Notably, all five methods predicted *MLH1* 1151 T > A (Val384Asp) to be pathogenic, and four out of the five methods predicted *BRCA1* 154C > T (Leu52Phe) to be pathogenic (Table 2).

**Table 2:** Prediction of the potential pathogenicity of nonsynonymous variants by using five algorithms

Gene	Mutation	Function	PROVEAN^a^	SIFT^b^	Polyphen2 HDIV^c^	Polyphen2 HVAR^d^	LRT^e^	MutationTaster2^f^
BRCA1	c.154C > T	p.Leu52Phe	N (−0.59)	D (0)	D (1)	D (0.985)	D	D (1)
BRCA1	c.1337G > T	p.Arg446Ile	D (−7.74)	D (0.007)	D (0.986)	D (0.930)	N	P (7.05e-05)
BRCA1	c.2566T > C	p.Tyr856His	D (−3.73)	D (0.015)	P (0.730)	P (0.733)	N	D (1)
MLH1	c.1151T > A	p.Val384Asp	D (−5.62)	D (0)	D (1)	D (0.998)	D	D (1)
MLH1	c.776T > C	p.Leu259Ser	D (−5.36)	D (0)	P (0.808)	P (0.706)	N	D (1)
MLH1	c.2135G > T	p.Trp712Leu	D (−7.28)	D (0.006)	P (0.576)	P (0.553)	D	D (1)

^a^PROVEAN score ≤ −2.5 was regarded as deleterious (D); score > − 2.5 was regarded as neutral (N);

^b^SIFT score ≤ 0.05 was regarded as deleterious (D);

^c^Polyphen2 HDIV score ≥ 0.957, probably damaging (D); 0.453 < Polyphen2 HDIV score < 0.957, possibly damaging (P);

^d^Polyphen2 HVAR score ≥ 0.909, probably damaging (D); 0.447 < Polyphen2 HVAR score < 0.909, possibly damaging (P);

^e^Deleterious (D), Neutral (N);

^f^Polymorphism (P), Disease causing (D)

## Discussion

In this study, we demonstrated the similarities in the genetic and epigenetic landscapes between patients with irregular menstrual cycles and ovarian cancer, providing further evidence to distinguish an irregular menstrual cycle as an independent risk for ovarian cancer. We showed that the DNA from ovarian tissues of patients with irregular menstrual cycles display global DNA hypomethylation, a hallmark characteristic of nearly all types of cancers [19]. We next showed that patients with irregular menstruation share miRNA and mRNA expression profiles similar to those observed in ovarian cancer, and that many oncogenic genes are upregulated in patients with ovarian cancer. Finally, we identified multiple point mutations in two genes that confer a significant increased risk for ovarian cancer, *BRCA1* and *MLH1*, in patients with irregular menstruation.

While these results begin to show a genetic relationship between ovarian cancer and irregular menses, they still do not illustrate whether these genetic changes directly lead to the phenotype of irregular menstruation, or if they are a product of abnormal hormone exposure as a result of irregular menstruation. Several studies have shown that aberrant hormone levels result in elevated risks for cancer, particularly sex hormones and the development and progression of gynecological cancers [20–24]. Furthermore, it is highly likely that a patient with irregular menstruation would have an atypical sex hormone profile. This warrants further investigation into the relationship of hormonal regulation and the genetic changes observed in patients with irregular menstruation.

We uncovered an appreciable array of genes with anomalous expression levels in patients with irregular menses. Many of these genes have established roles in oncogenesis with clearly defined pathways. Several of these genes are involved in pathways that define the hallmarks of cancer, such as *VEGFC* (lymphangiogenesis), *CASP2* (apoptosis and cell stress), and *CDC20* (cellular proliferation) [25–27]. To fully appreciate the effect of aberrant gene expression in the ovarian tissue of patients with irregular menstruation, future studies investigating the activity of the molecular genetic pathways involved with these genes are warranted.

In our identification of point mutations in the *BRCA1* of patients with irregular menstruation, we showed that one of the *BRCA1* variants resulted in a significant change in the three-dimensional confirmation of the final protein product of the gene. The three point mutations in the BRCA1 gene were further checked in the Human Gene Mutation Database (http://www.hgmd.cf.ac.uk). To date, these mutations have not been reported to be associated with ovarian cancer, but c.154C > T and c.2566T > C may be involved in the progression of breast cancer. Therefore, further investigation into the activity of these variants and their downstream effectors is needed to confirm their physiological significance. Notably, many BRCA1 variants have unknown clinical significance [28]. In addition, recent evidence suggests a significant increase in the frequencies of BRCA1 polymorphisms in patients with PCOS compared to controls [29]. However, these data are only preliminary evidence, and we cannot recommend that most patients with PCOS and irregular menstruation get tested for BRCA1, but BRCA1 testing and familial risk assessment may be more desirable for people with a family history of PCOS or ovarian cancer.

## Conclusion

Here we further distinguished irregular menstruation from PCOS as an independent risk factor for the development of ovarian cancer. We show that the ovarian tissue of patients with irregular menstruation displays DNA hypomethylation and shares miRNA and mRNA profile similarities with ovarian cancer. We also identified multiple point mutations in two genes that confer a significant increased risk for ovarian cancer in patients with irregular menstruation.

## Additional files

Additional file 1: Primers used in this study. (PDF 26 kb)

Additional file 2: Information of 46 normal ovary tissue samples downloaded from the GEO database. (PDF 56 kb)

## Abbreviations

CGI: CpG islands
GEO: Gene Expression Omnibus
LINE-1: Long interspersed nuclear element-1
miRNAs: microRNAs
PCOS: Polycystic ovary syndrome
SRA: NCBI Sequence Read Archive
TCGA: The Cancer Genome Atlas
