# Supplementary material for: Genetic and epigenetic characteristics in ovarian tissues from polycystic ovary syndrome patients with irregular menstruation resemble those of ovarian cancer
Source: BMC Endocr Disord. 2019 Mar 12;19:30. doi: 10.1186/s12902-019-0356-5 (PMC6416936; doi:10.1186/s12902-019-0356-5)
Supplement: Supplementary file 1 — Primers used in this study. (PDF 26 kb) [file 12902_2019_356_MOESM1_ESM.pdf]

Additional file 1. Primers used in this study.

Gene	Forward primer	Reverse primer
ACLY	GTGTGGACGTGGGTGATGTG	TTTGATGTCCTCAGGATTCAGTTTC
ADM	ATGAAGCTGGTTTCCGTCG	GACATCCGCAGTTCCCTCTT
ANGPT1	GTGCTCACGTGGCTCGACTATA	GCTCAGCAGTTTCTTCCCTTTTT
ANGPT2	TTTACTCTGAGCTGTGATCTTGTCTTG	GACCTGATATTGCTTCTTTCCTATGC
ARNT	CTGCCAACCCCGAAATGACAT	CGCCGCTTAATAGCCCTCTG
ATP5A1	ATGACGACTTATCCAAACAGGC	CGGGAGTGTAGGTAGAACACAT
AURKA	GAGGTCCAAAACGTGTTCTCG	ACAGGATGAGGTACACTGGTTG
BCL2L11	CCCAAGAGTTGCGGCGTAT	ACATGACAACTACACGACAACTTCCT
BIRC3	AGCTTGCAAGTGCGGGTTT	CCACCATCACAGCAAAAGCA
BMI1	CTTGGCTCGCATTCATTTTCT	CACATCAGGTGGGGATTTAGC
CA9	TTTGCCAGAGTTGACGAGGC	GCTCATAGGCACTGTTTTCTTCC
CASP2	AGCTGTTGTTGAGCGAATTGT	AGCAAGTTGAGGAGTTCCACA
CASP7	TGGAAAATGGAGCCATGACAA	AAGGTATTCTCTTATCTGTCTCAGCTC ACT
CASP9	AAGCCCAAGCTCTTTTTCATC	ACTCGTCTTCAGGGGAAGTG
CCL2	CAGCCAGATGCAATCAATGCC	TGGAATCCTGAACCCACTTCT
CCND3	TACCCGCCATCCATGATCG	AGGCAGTCCACTTCAGTGC
CDC20	GCACAGTTCGCGTTCGAGA	CTGGATTTGCCAGGAGTTCGG
CFLAR	TCAGCCTTCCAAGATAGATACTCCAT	AACCCACGTGGATCATCTATGC
CPT2	CATACAAGCTACATTTCGGGACC	AGCCCGGAGTGTCTTCAGAA
DDB2	ACCTCCGAGATTGTATTACGCC	TCACATCTTCTGCTAGGACCG
DDIT3	GGAAACAGAGTGGTCATTCCC	CTGCTTGAGCCGTTCATTCTC
DKC1	GCTAAGTTGGACACGTCTCAG	TGCAAGAGGTGTATAGTGTGTTG
E2F4	ATCGGGCTAATCGAGAAAAAGTC	TGCTGGTCTAGTTCTTGCTCC
ERCC3	CTAACTGCCTACTCCTTGTATGC	TCCATAGCTGACAGTACACAACT
ERCC5	TGCAGTCCGTCGTAGAAGAATT	GAGCCGATGAAACAAAGTGAGA
FASLG	CCCATTTAACAGGCAAGTCCAA	CCAGAAAGCAGGACAATTCCAT
FGF2	AGTGTGTGCTAACCGTTACCT	ACTGCCCAGTTCGTTTCAGTG
FOXC2	CGCCTAAGGACCTGGTGAAG	CCATGATGAACTGGTAGATGCC
G6PD	CTTTGCCCGCAACTCCTATG	GGCATTCATGTGGCTGTTGA
GADD45G	CAGATCCATTTTACGCTGATCCA	TCCTCGCAAAACAGGCTGAG
GPD2	CACCAGAGGACTAAAAACAGCC	GGAGCTGATAAATGGGGAGCA
GSC	CCTCCGCGAGGAGAAAGT	CGTTCTCCGACTCCTCTGAT
HMOX1	CAGCTGTCTCAAACCTCCAAAAG	AGTGGTCATGGCCGTGTCA
IGFBP3	AGCCAGCGCTACAAAGTTGACT	GCTTGGACTCGGAGGAGAAGTT
IGFBP5	ACCTGAGATGAGACAGGAGTC	GTAGAATCCTTTGCGGTCACAA
KDR	CACCACTCAAACGCTGACATGTA	CCAACTGCCAATACCAGTGGAT
KRT14	GTGGTGATGGGCTTCTGG	CCATTGATGTCGGCTTCC
LDHA	TTGACCTACGTGGCTTGGAAG	GGTAACGGAATCGGGCTGAAT
LIG4	AGCAAAAGTGGCTTATACGGATG	TGAGTCCTACAGAAGGATCATGC
LPL	TCATTCCCGGAGTAGCAGAGT	GGCCACAAGTTTTGGCACC
MAPK14	TGGATTTTGGACTGGCTCG	CATGGCTTGGCATCCTGTTA
MCM2	ATGGCGGAATCATCGGAATCC	GGTGAGGGCATCAGTACGC
NOL3	GACCGCAGCTATGACCCTC	CTCCGGTTCAGCCTCTTTAGA
OCLN	AGGAACCGAGAGCCAGGT	TGAGCAATGCCCTTTAGCTT
PFKL	GGTGGGTTTGAGGCCTATGAA	GGGACGTTGTTGCTGATGGT
PGF	GAACGGCTCGTCAGAGGTG	ACAGTGCAGATTCTCATCGCC

PINX1	CCAGCGGATGCTAGAGAAGAT	CGAGTCCCAGGTGGTTATTTTTC
PPP1R15A	ATGATGGCATGTATGGTGAGC	AACCTTGCAGTGTCCTTATCAG
SERPINF1	TTCAAAGTCCCCGTGAACAAG	GAGAGCCCGGTGAATGATGG
SKP2	ATGCCCCAATCTTGTCCATCT	CACCGACTGAGTGATAGGTGT
SNAI1	TCGGAAGCCTAACTACAGCGA	AGATGAGCATTGGCAGCGAG
SNAI2	CCATTCCACGCCCAGCTA	TCACTCGCCCCAAAGATGAG
SNAI3	ACTGCGACAAGGAGTACACC	GAGTGCGTTTGCAGATGGG
SOD1	GCTGGTTTGCGTCGTAGT	TTCTGCTCGAAATTGATG
SOX10	CCTCACAGATCGCCTACACC	CATATAGGAGAAGGCCGAGTAGA
STMN1	TCAGCCCTCGGTCAAAAGAAT	TTCTCGTGCTCTCGTTTCTCA
TBX2	CACGGCTTCACCATCCTAAAC	TGCGGAAGGTGCTGTAAGG
TEK	TTAGCCAGCTTAGTTCTCTGTGG	AGCATCAGATACAAGAGGTAGGG
TERF1	TGCCGACCCTACTGAGGAG	GCAGAGGAAATCGAGCATCCA
TERF2IP	TGAAGGACCGCTACCTCAAG	GGCTTCCACAAGCATCTTTTTG
TINF2	TTGGAGGCACAGGAAACTTTT	AGGGTTCCCCATACTCTTGTTC
TNKS2	CAACTGCCACGCAAGTGATG	AGAGGAGTGAATTGCCACAAG
UQCRFS1	ACCCCTGTTTGTGCGTCATAG	AAGGGCAGTAATAACCACCAAAA
VEGFC	CTCTCAAGGCCCCAAACCA	GACATGCATCGGCAGGAAGT
WEE1	AACAAGGATCTCCAGTCCACA	GGGCAAGCGCAAAAATATCTG
XIAP	ACCTGCAGACATCAATAAGGAAGAA	ACCGCACGGTATCTCCTTCAC
GAPDH	ACGGATTTGGTCGTATTGGG	CGCTCCTGGAAGATGGTGAT
