# Supplementary material for: Genetic and epigenetic characteristics in ovarian tissues from polycystic ovary syndrome patients with irregular menstruation resemble those of ovarian cancer
Source: BMC Endocr Disord. 2019 Mar 12;19:30. doi: 10.1186/s12902-019-0356-5 (PMC6416936; doi:10.1186/s12902-019-0356-5)
Supplement: Supplementary file 2 — Information of 46 normal ovary tissue samples downloaded from the GEO database. (PDF 56 kb) [file 12902_2019_356_MOESM2_ESM.pdf]

Additional file 2. Information of 46 normal ovary tissue samples downloaded from the GEO database.

Rank	GSE series number	GSM number for samples	Samples counts	Platform
1	GSE18521	GSM462643, GSM462644, GSM462645, GSM462646, GSM462647, GSM462648, GSM462649, GSM462650, GSM462651, GSM462652	10	Affymetrix Human Genome U133 Plus 2.0 Array
2	GSE27651	GSM372258, GSM372259, GSM372260, GSM372261, GSM372262, GSM372263	6	Affymetrix Human Genome U133 Plus 2.0 Array
3	GSE34405	GSM848593, GSM848594, GSM848595	3	Affymetrix Human Genome U133A Array
4	GSE38666	GSM947277, GSM947278, GSM947279, GSM947280, GSM947281, GSM947282, GSM947283, GSM947284, GSM947285, GSM947286, GSM947287, GSM947288	12	Affymetrix Human Genome U133 Plus 2.0 Array
5	GSE36668	GSM898305, GSM898306, GSM898307, GSM898308	4	Affymetrix Human Genome U133 Plus 2.0 Array
6	GSE43346	GSM1060767	1	Affymetrix Human Genome U133 Plus 2.0 Array
7	GSE1133	GSM18838, GSM18839, GSM18997,	4	Affymetrix Human Genome U133A Array

		GSM18998		
8	GSE2361	GSM44674	1	Affymetrix Human Genome U133A Array
9	GSE105437	GSM2825980, GSM2825981, GSM2825982, GSM2825983, GSM2825984	5	Affymetrix Human Genome U133 Plus 2.0 Array
Total			46	
